# Basic microsurgical skills: suturing

**Published:** 2023-12-01

**Authors:** Rebecca Jones, William Dean

**Affiliations:** 1 Ophthalmology Registrar: Cheltenham General Hospital, Cheltenham, UK.; 2 Assistant Clinical Professor: ICEH, LSHTM, UK. Honorary Associate Professor: University of Cape Town, South Africa.; 3 Consultant: Speciality Director, Gloucestershire Hospitals NHS Foundation Trust, UK.

**Operating under magnification is a challenging but vital skill that all ophthalmologists must develop.**

From repairing an open globe, to suturing a rectus muscle, or closing a trabeculectomy flap, basic suturing using a microscope (or loupes) is an essential skill.

## The tools of microsurgery

**Figure 1:**
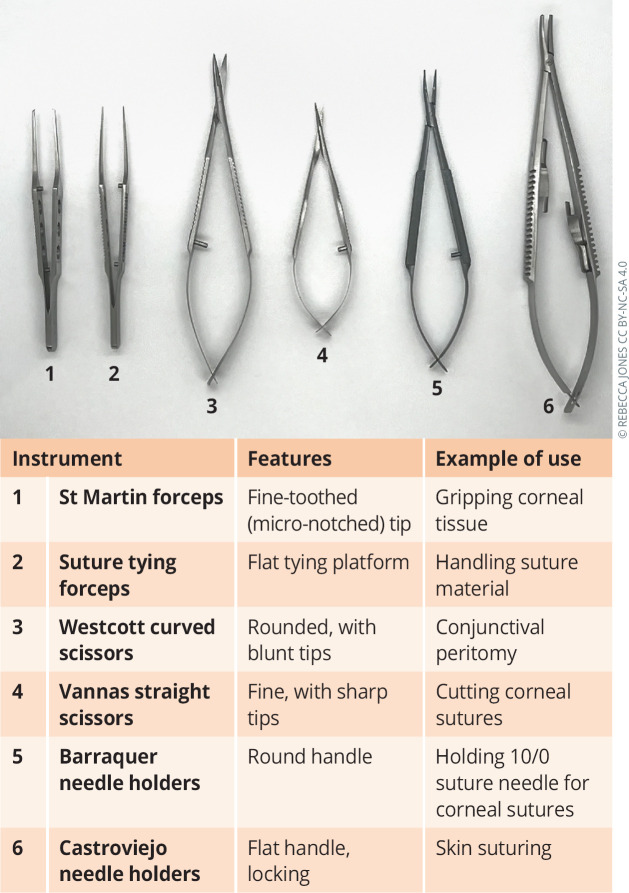
A basic set of instruments used for ophthalmic microsuturing. From left to right: St Martin forceps, suture tying forceps, Westcott curved scissors, Vannas straight scissors, Barraquer needle holders, and Castroviejo needle holders.

Since ocular tissues are so delicate, it is important to use instruments safely and to select the correct instrument for each task (Figure 1). For example, toothed forceps are designed for grasping tissue, while non-toothed forceps have a tying platform that enables the surgeon to grip suture material.

**Figure 2:**
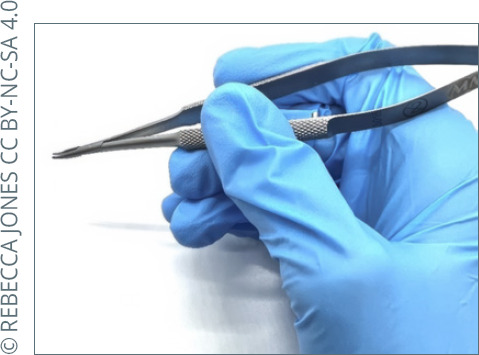
Hold surgical instruments as you would hold a pen or pencil.

Instruments are usually held as a pen would be (Figure 2). Fine movements are made using the fingers and wrist, with the hand resting to stabilise the motion.^1^

Sutures vary by needle type and suture material. Choice of suture depends on the type of tissue, strength and duration of wound support required. There are absorbable and non-absorbable sutures, which may be monofilament or multifilament (braided). Common sutures include:

- Vicryl (polyglactin), an absorbable suture which provides tissue support for 21 days, but induces inflammation as it is absorbed by hydrolysis reaction
- Ethilon (nylon), a non-absorbable suture, which would need removal if no longer required to support wound healing
- Prolene (polypropylene), also a non-absorbable suture.

It is generally best to use the thinnest suture suitable to accomplish the task. For example, 10-0 is preferable for corneal suturing, 8-0 for conjunctival suturing, and 6-0 for skin or tarsal plate suturing and reattaching rectus muscles.

The type of suturing needle can vary as follows:

- By cross-section of the needle, which affects the cutting planes of the needle
- By curvature arc of the needle, with the 3/8 circle needle being most commonly used.

**Corneal gluing**

Occasionally, corneal gluing may be used as an alternative to suturing. However, avoid gluing if watertight closure cannot be achieved – suturing will be needed instead. Use a cyanoacrylate or fibrin glue.

## Corneal suturing

Corneal suturing is a core microsurgical skill. In low- and middle-income countries, it is usually one of first types of microsurgical suturing skills many trainees will learn. Corneal suturing is needed to repair traumatic corneal lacerations and, on occasion, cataract surgery incisions.

A 10/0 nylon suture is usually used for corneal suturing. The suture needle must be handled with care, as grasping the tip will blunt the needle, and grasping the swage may cause the suture material to detach from the needle. Further detail on the anatomy of the suture and handling the needle safely can be found in the article on passing sutures, in this issue.

**Figure 3:**
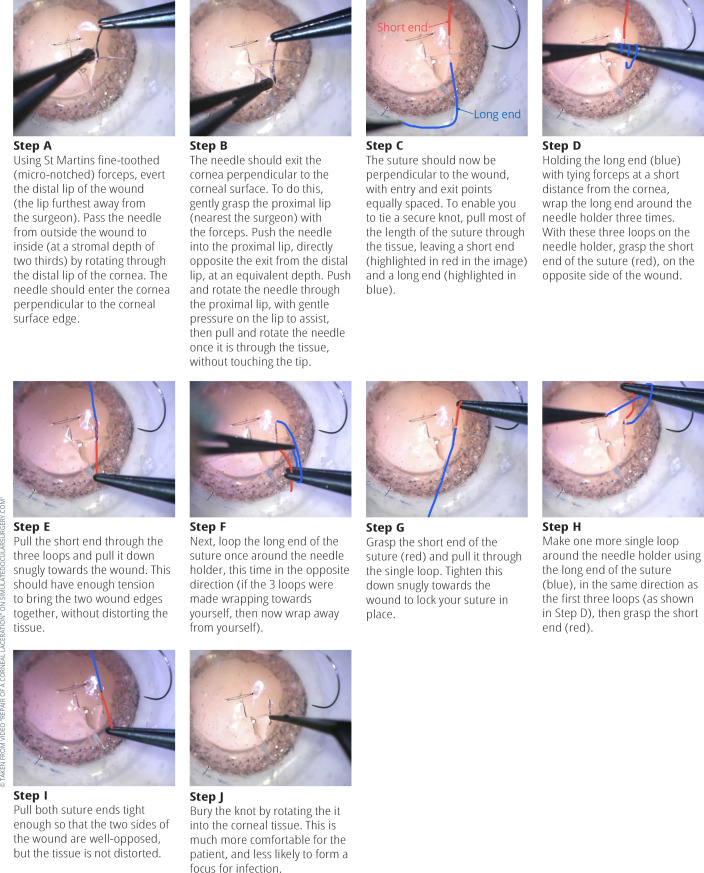
The 3-1-1 technique for interrupted sutures

**Interrupted sutures** are the first type a trainee surgeon is likely to learn. The 3-1-1 technique described in Figure 3 produces a firm knot that is small enough to be buried by rotating the knot into the corneal tissue. This is more comfortable for the patient, and less likely to form a focus for infection. **Note:** when making more than one interrupted suture, ensure they are equally spaced and of equal length.

The images used to illustrate these steps were adapted from the video ‘Repair of a corneal laceration’ on www.simulatedocularsurgery.com,^1^ and show a surgeon practising interrupted corneal suturing on an artificial eye.

## Practice, practice, practice

**Figure 4:**
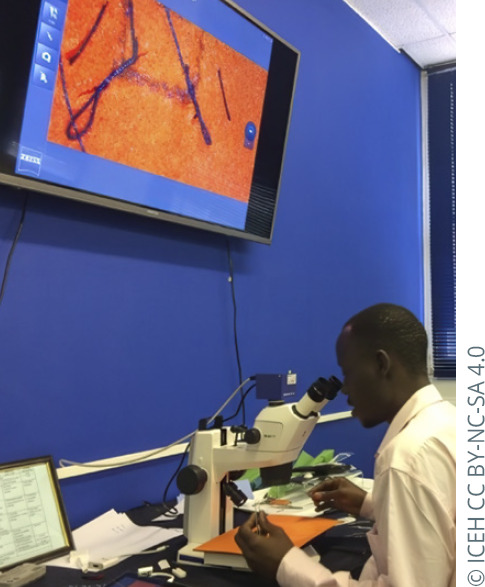
Ophthalmic trainees practicing suturing on foam sheets

Each of these steps can be perfected in a simulated environment, prior to operating on a patient. Operating microscopes or portable training microscopes may be used to practise corneal suturing (Figure 4).^2^ Model eyes are available to practise these skills, but foam (even that used in the suture packaging) can also be a good medium to simulate corneal tissue. If a microscope is not available, the same suturing techniques can be practised on a larger scale using 7-0 sutures and the naked eye.

Eyelid laceration repair can be performed without use of a microscope. Silicone suturing pads or fruit (such as banana peel) may be used to replicate eyelid tissue. The use of animal models has also been validated, for example Pfaff's use of a split pig head model to teach eyelid margin repair.^3^ Guidance for practicing eyelid closure using simulation is also available on: http://oculoplastics.info/video/thebasics/eyelid-margin-closure/

## Feedback

Simulation practice can be enhanced by peer or supervisor feedback, in addition to self-reflection using assessment rubrics. A useful tool called the Ophthalmic Simulated Surgical Competency Assessment Rubric (OSSCAR) has been created to assess various ophthalmic procedures, including corneal laceration repair.^4^ By comparing their attempt to the assessment rubric, the trainee surgeon can identify areas for improvement. This may be augmented by video recording their practice sessions.

Beginning with simple linear corneal lacerations, the complexity of surgical techniques can be increased by creating irregular, shelved or stellate lacerations and by practising both right- and left-handed. Once interrupted sutures are mastered, trainees may progress to butterfly, purse-string or continuous sutures.

**Useful videos**

These videos all demonstrate a good technique for corneal suturing.

**Corneal suturing, by Derek Ho**

How to suture a corneal laceration, demonstrating 3-1-1 and slipknot techniques, on a model eye. https://bit.ly/Corneal_Ho

**How to suture a phaco incision, by Cataract Coach.com**

Video of live surgery demonstrating how to suture a cataract surgery incision using a 10-0 nylon suture. https://bit.ly/47bhmjL

**Keys to Corneal Suturing, by Christopher J Rapuano**

Video demonstrating key tips for corneal laceration repair, using a pig's eye. https://bit.ly/3MKzAAr
